# Tubectomy of Pregnant and Non-pregnant Female Balinese Macaques (*Macaca Fascicularis*) With Post-operative Monitoring

**DOI:** 10.3389/fvets.2021.688656

**Published:** 2021-09-09

**Authors:** Stefan Deleuze, Fany Brotcorne, Roland Polet, Gede Soma, Goulven Rigaux, Gwennan Giraud, Fanny Cloutier, Pascal Poncin, Nengah Wandia, Marie-Claude Huynen

**Affiliations:** ^1^Research Unit FARAH, Equine and Companion Animal Reproduction Pathologies Clinic, Veterinary Medicine Faculty, University of Liège, Liège, Belgium; ^2^Research Unit SPHERES, Department of Biology, Ecology and Evolution, Sciences Faculty, University of Liège, Liège, Belgium; ^3^Primate Research Center, Veterinary Medicine Faculty, Udayana University, Denpasar, Indonesia; ^4^Pan Anima, Tournai, Belgium; ^5^Research Unit FOCUS, Department of Biology, Ecology and Evolution, University of Liège, Liège, Belgium

**Keywords:** bipolar thermocautery, laparoscopy, monkey, population control, salpingectomy, sterilization, wildlife

## Abstract

Worldwide, primates, and humans increasingly share habitats and often enter in conflict when primates thrive in human-dominated environments, calling for special management measures. Reproductive control is increasingly used to manage population growth but very few monitoring data are available. Therefore, the efficiency and implications of such programs require a careful examination. In the context of a contraception program in wild female long-tailed macaques in Ubud, Bali, conducted over four successive campaigns between 2017 and 2019, including 140 females (i.e., 41.9% of the reproductive females of the population in 2019), modifications of an endoscopic tubectomy procedure, a permanent sterilization method, clinical evaluation of this method, and the post-operative monitoring results of the neutered females after release are described. This surgical approach was applicable for pregnant females: 28.6% of the treated females were pregnant at the time of the surgery. The procedure used a single lateral port to reach and cauterize both oviducts in non-pregnant as well as in early to mid-term pregnant females. Pregnant females nearer to term required a second lateral port to access both oviducts masked by the size of the gravid uterus. Moreover, bipolar thermocauterization was utilized successfully without resection to realize the tubectomy. The average duration of the laparoscopic surgery was 14 min for non-pregnant females and 22 min for pregnant females. Animals were released 3 h 22 min in average following their capture. This short holding time, recommended for free-ranging primates, was made possible by the minimal invasiveness of the sterilization approach. A laparoscopic post-operative evaluation conducted on two patients during the following campaign confirmed that the oviducts were definitely disrupted and no longer patent. Moreover, no new pregnancies in sterilized females were recorded during the 3-year observation period. The survival rate of the treated females 6 months after sterilization was high (96.3%) with no major post-operative complications clinically recorded. Among females that were pregnant during surgery, 81.1% were confirmed to experience term delivery. This study demonstrates the safety and efficiency of endoscopic tubectomy, even for pregnant females, as a mean of wild macaques' population control.

## Introduction

Worldwide, a growing proportion of primate populations of various species thrive in human-modified habitats (1). As a consequence of the rapid land-use change, these populations are increasingly forced to cohabit with humans (2). The macaques (*Macaca* sp.) in particular share one of the widest spatial overlaps with humans, and sometimes proliferate, even in urban environments (3). This is the case for the long-tailed macaque (*Macaca fascicularis*) which increasingly gravitates toward human environments throughout south-east Asia, becoming sometimes locally overabundant within human-dominated lands (4). Uncontrolled increase of populations in urban or peri-urban settings often causes conflicts between macaques and people, threatens public health, multiplies nuisance problems for people, and has in turn significant impacts for macaques (2, 5). Regarding sanitary risk, non-human primates present a high potential for zoonotic pathogen exchange with humans by their close phylogenetic relationship (6), especially when they expand their range in anthropogenic landscape and increase in abundance (7). The risk of infectious disease outbreaks increases in human-macaque interface zones with intensified inter-species contacts, with implications for both public health and primate conservation (8). The recent COVID-19 pandemic events have recalled for a pressing need to monitor these populations at the interface with humans (9). Managing these escalating conflicts has to consider altogether macaque population sustainability, public health implications, as well as cultural and economic concerns of neighboring local communities (10).

In several places in Asia, measures have been carried on to limit the expansion of monkey overpopulation associated with human-macaque conflicts (3). Contraception represents an ethical alternative to culling and translocation and is increasingly used in free-ranging populations. Over the years, various approaches have been used including male castration (11), progestin-based contraceptive treatment administered to females (12), programs combining immune-contraception on females and neutering chemical vasectomy on males (13), or endoscopic tubectomy as a permanent sterilization method for females. The latter technique has since been used either alone (14) or combined with vasectomies (15–17) on a large-scale on rhesus macaques (*Macaca mulatta*) in different sites in India and Hong Kong.

Although wildlife fertility control has gained influence over the past decades (18), experimental data are critically lacking in this field when it comes to free-ranging primates. This situation urges conducting systematic studies during and in the aftermath of the fertility control interventions to monitor the impacts, efficiency, and adequacy of the methods adopted with primates (10, 19). As for laparoscopic tubectomy, clinical intra-, and post-operative evaluations have been described in a small number of rhesus (17) and Formosan macaques (*M*. *cyclopis*) (20) in captive settings, and more recently in a long term program in field conditions in free-ranging rhesus cross long-tailed macaques (14). However, the application of laparoscopic tubectomy to *M. fascicularis* in free-ranging settings, its clinical evaluation and possible modification to optimize the technique as well as a long-term post-surgery monitoring remain lacking and need to be documented. Reasons for selecting this technique with urban long-tailed macaques are manyfold but most of all, the species' reproductive biology [i.e., a multimale/multifemale mating system where a female is likely to mate with multiple males; (21)] and social dynamics [i.e., a female philopatry system of resident matrilineal groups wherein demographic recruitment is a function of births and male migrations; (22)], naturally orient the choice toward female-based sterilization.

The first objective of this study is to document the modification of the endoscopic tubectomy procedure used in the long-tailed macaque, to assess its applicability with gravid females, and to provide a clinical evaluation of the method and its potential associated complications. The second objective is to assess the suitability of this sterilization technique in a free-ranging setting via a long-term post-op monitoring of the contracepted females after release as regards to survival and births.

## Materials and Methods

### Study Site

**Figure 1:**
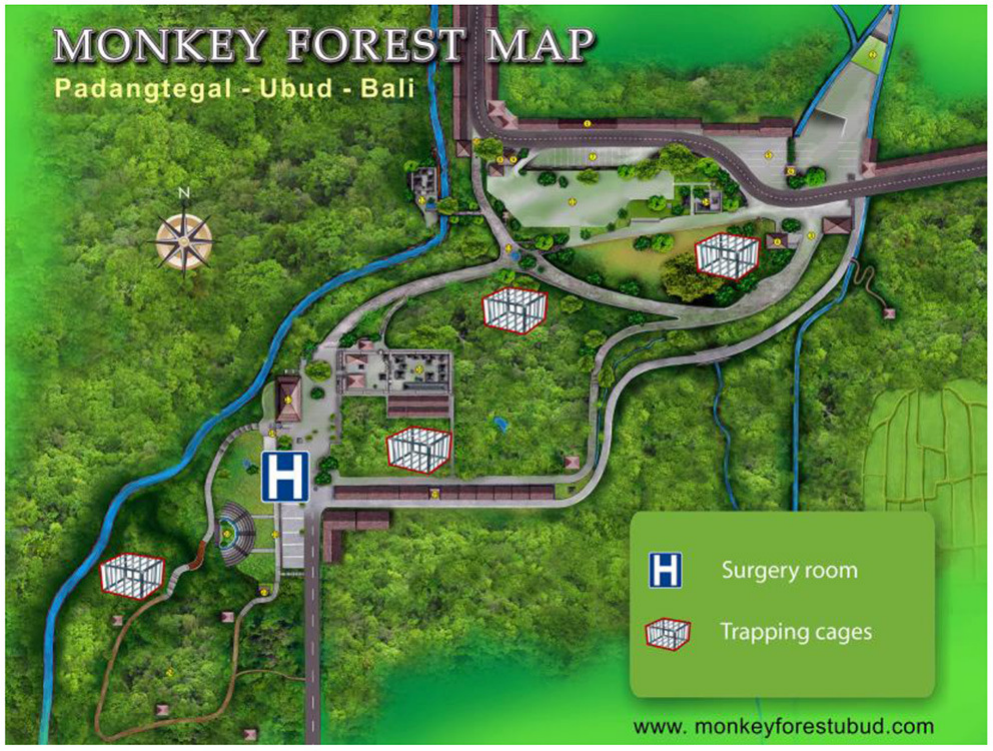
The study site and location of the four trapping cages and the surgery room (modified from www.monkeyforestubud.com).

This research was conducted in the Ubud Monkey Forest (*Mandala Wisata Wenara Wana*), a forest sanctuary located in Ubud in south-central Bali (8°31′S-155°15′E), Indonesia. It is a highly frequented tourist site, visited by more than one million of tourists per year, generating a plethora of commercial activities and economic incomes (23). The site consists of a Hindu temple complex within a 21 ha of secondary mixed forest enclosed in an urban landscape (Figure 1). This sanctuary shelters both a long-term residential and free-ranging population of long-tailed macaques. The village committee has managed this site since 1980 and provisions the macaques on a daily basis with sweet potatoes and various fruits and vegetables.

### Study Population and Local Issue

Despite the limited size of available habitat (~21 ha in 2018), the population of macaques in the Ubud Monkey Forest dramatically grew over 30 years, with a 10% mean annual growth rate (24). In 2017, the population counted 754 individuals split into seven social groups (C, X, S, N, H, T, L) and kept increasing up to 1,059 individuals in 2019, reaching the highest population density (50 individuals/ha) reported for that species in the semi-wild (25). Reasons for this constant growth and high density include the combined effects of forest loss and limitation to emigration imposed by the recent development of human barriers (26), the absence of natural predators, the cultural protection the macaques granted in the temple forest site, and the regular large-scale food provisioning of the monkeys which otherwise would not find enough resources in the forest to sustain themselves (27). Therefore, the site is now overcrowded and this situation imposes an array of detrimental consequences and costs to the macaques, including growing social tensions, increased aggressions and fading social group cohesiveness (28). Concurrently, the stakeholders of the sanctuary report increasing complaints from the neighborhood about macaque nuisances as well as aggressive interactions between macaques and visitors of the site.

In the aforementioned conditions, the question of population control has become crucial and a sterilization program has been initiated in 2017 as a means of controlling the density of monkeys in an ethical way. This first required the careful estimation of the magnitude of the sterilization needed. A stochastic matrix population model (29) based on a 10-year census dataset of the population was implemented to estimate the sterilization proportion of reproductive females required to achieve population stability (i.e., a null population growth). Simulations suggested that 47% of the reproductive females in 2017 (i.e., 135 individuals) had to be sterilized in order to reach population stability (30). By 2019, 41.9% of the reproductive females were sterilized. A demographical monitoring and further projections are in progress to check the model suitability and calibrate the needed adjustment campaigns (31).

### Study Animals

Over successive sterilization campaigns between 2017 and 2019, 140 reproductive females underwent an intervention for tubal cauterization. Only reproductive females (identified by the presence of nipples development and sexual skin swelling) were selected for surgery. These reproductive females were either full adults or subadults, identified on a combination of morphological characteristics including dental eruption patterns (32) and development stage of sexual organs (33, 34). Subadults (2.5–3.5 years) are nulliparous females characterized by buttonlike nipples and 24–28 permanent teeth. Adults are primiparous or multiparous females with elongated nipples and 28–32 permanent teeth. Out of 140 females, 129 (92.1%) were adult and 11 (7.9%) were subadults. Characteristics of the treated animals are documented in Supplementary Table 1.

### Procedure Description

#### Capture and Anesthesia

The capture protocol complies with the ethical recommendations of Jolly et al. (35) for capturing free-ranging habituated primates. The capture-release process was conducted by the trained local staff of the Ubud Monkey Forest. Four giant trapping cages (5 mL × 2.4 mW × 2 mH) located within the groups' home range were baited daily with food since 2016 to habituate the monkeys to use the cage (Supplementary Figure 1). The cages were equipped with a trapdoor that had to be manually maneuvered by an operator at distance. On capture days, once an acceptable number of reproductive females had entered the cage, the trap door was closed and another operator slid a movable partition on the back side of the cage to lead the monkeys into smaller squeeze cages for anesthesia (Supplementary Figure 2). Darting using blowpipe was occasionally used to increase numbers of females and reach a minimum of 8–10 surgeries per day. The first dose administrated was based on the visual assessment of the age class. Based on previously collected data, estimated bodyweights of adult and subadult females were 5.5 and 3.5 kg, respectively. Animals were induced using intramuscular injection of 5 mg/kg of ketamine (Ketamine 10%®, Kepro, Holland) and 1.33 mg/kg of xylazine (Xyla®, Interchemie, Holland). Once induced all animals were weighed to allow adjusted dosage of all drugs administered thereafter. For animals that underwent surgery, anesthesia was then maintained with intermittent intravenous boluses of 1% propofol (Recofol®, 1 mg/kg, Primex, Switzerland). Vital parameters, respiratory and heart rate, pulse oximetry (with the probe placed either on the cheek, tongue, or tip of the index finger) and rectal temperature were monitored all along the procedure. Given that the capture method used a food-baiting strategy, the animals were not fasted before their anesthesia. After anesthesia, monkeys were equipped with a pediatric bracelet marked with an individual code for ID tracking and were transported to an air-conditioned operation building located inside the sanctuary (Figure 1).

#### Clinical Exam and Preparation

A general clinical exam was conducted to care for potential injuries, remove food material from the oral cavity and cheek pouches to reduce the risk of aspiration pneumonia, and assess the position and size of the uterus that enlarged cranially with pregnancy for subjective staging via abdominal palpation (early stage defined as <1.5 months, mid-term as 1.5–3 months, and advanced stage as >3 months). Females selected for surgery were catheterized in the cephalic vein for fluidotherapy with saline perfusion (NaCl 0.9%) and received a dose of antibiotic (Betamox LA®, amoxicillin, 15 mg/kg, I.M., Norbrook, United Kingdom), non-steroidal anti-inflammatory (Tolfedine® CS, 4 mg/kg, subcutaneous, Vétoquinol, France), and eye lubricant (Visine®, Johnson & Johnson) to protect the cornea from desiccation. The abdomen was shaved from the xiphoid appendix to the pubis and as far as the lower line of the flank. Patients were placed in dorsal recumbency with the pelvis placed on a cushion to slightly elevate it and allow movement of the abdominal organs cranially in the abdominal cavity (Trendelenburg position). The surgical site was scrubbed with antiseptic soap and 70% alcohol and draped.

#### Surgery

The tubectomy procedure routinely consisted in a laparoscopic two-5 mm-ports approach. Briefly, a 15 mm skin incision was performed with a scalpel blade ~1–2 cm cranially to the umbilicus to allow placement of a Veress needle (150 mm, Covidien^TM^, USA), taking care to avoid the underlying organs (e.g., the gravid uterus) or any blood vessel. The abdomen was then insufflated with CO_2_ to a pressure of 10–12 mmHg (Insufflator, WISAP®, Germany) until a satisfactory abdominal distension was achieved. The Veress needle was then removed and a 5 mm trocar was inserted through the same skin incision. The rigid telescope (Hopkins® straight 0°, 5 mm, 29 cm, Karl Storz, Germany), connected to an endoscopic camera (Sopro-Comeg 368 HD, Germany) and light source (LED Nova 150, Karl Storz, Germany) was introduced in the abdomen via the cannula. The abdominal cavity was evaluated for topography of the reproductive organs, gravidity and presence of adhesions. The site for the insertion of a second 5 mm trocar was localized in the left lower quadrant, just cranially to the oviduct. After a skin incision of ~5 mm, the second trocar was inserted under visual endoscopic control, guided by transillumination thus easily avoiding large blood vessels. The ampulla of the left oviduct was easily recognized as flexuous, and paler than the rest of the organ. Using a bipolar Kelly grasping forceps (RoBi® 38151, Karl Storz, Germany) connected to a thermo-cauterization unit (Autocon® II 80, Karl Storz, Germany), it was grasped and coagulated until blanching of the entire oviductal segment was observed. A second cautery was performed proximally to the first. The ampulla of the right side was accessed through the same port and cauterized in a similar way (Supplementary Video 1), except for pregnant females nearer to term for which a third access in the right flank was performed to reach the right oviduct (Supplementary Video 2). The sites of thermo-cauterization were checked for adequate cauterization of the sections of the oviduct as they were not resected and were left in place. The reproductive organs and the rest of the abdominal cavity were carefully evaluated for any sign of bleeding or complication. The telescope and cannulae were removed and the abdomen was deflated by gentle massage. The two abdominal incisions were closed using 3–0 polyglecaprone (Monocryl®, Ethicon, USA) in simple interrupted pattern. An intradermal suture with the same material was used for the skin incisions (Supplementary Figure 3).

Anesthesia charts including time of drug administration, duration of surgery from incision to suture, and from induction to release, as well as clinical remarks were recorded.

#### Marking and Release

A permanent double marking for sterilized females was used with a microchip transponder inserted in the left antebrachium and a tattoo on their chest to permit remote identification (Supplementary Figure 4). After marking, the identifying pediatric bracelet was removed, a reversal, atipamezole hydrochloride (Antimedin®, 0.02 mg/kg, Dong Bang Co., Korea) was administrated intramuscularly (using a volume consistent with that of the previously administered xylazine) and animals were positioned in lateral recumbency in individual holding cages for recovery (Supplementary Figure 5). Animals were monitored for signs of pain during the recovery period. When they showed clear signs of full recovery from anesthesia (i.e., strong grip and climbing reflex, visual fixation, and following reflexes), individuals were transported in their holding cage to the capture site and released in their group.

#### Post-operative Monitoring

This study has been conducted over 3 years (2017–2020) during four successive sterilization campaigns (16 days in total: 3 days in July 2017 and July 2018, 5 days in February 2019 and August 2019). In parallel with the campaigns, a long-term monitoring of the contracepted females was conducted. Those females were monitored over 27 months split in several field observation periods between 2017 and 2020 (i.e., July 2017–September 2018, February 2019–September 2019, and December 2019–Mars 2020). Within 6 months after their surgery, the general body condition of contracepted females in the field, as well as any injury on sutures or elsewhere, disabilities, or cues indicating major unhealthy conditions were assessed. For the females pregnant at the time of the surgery, information about the pregnancy process and births was collected. Delivery failure in contracepted pregnant females was defined as abortion and perinatal mortality (36), based on clinical observation of (non-)occurrence of births after the intervention. Finally, survival of the sterilized females was defined as individuals still present at least 6 months after surgery.

## Results

Over the four sterilization campaigns, 140 long-tailed female macaques (129 adult and 11 subadult females) have been treated and monitored (Supplementary Table 1). The average weight of these females was 5.2 kg (*N* = 140). Adult females weighed more (5.3 kg, *N* = 129) than subadults (4.0 kg, *N* = 11).

### Intraoperative Evaluation

#### Success of Tubectomy and Pregnancy Stage-Related Feasibility

Among the 140 treated females, 136 (97.1%) were successfully tubectomized. Forty of these females (28.6%) were at various stages of pregnancy (early stage: 7.9%, mid-term: 7.1%, advanced stage: 13.6%). Among these 40 pregnant females, 3 (7.5%) could not be successfully sterilized due to various intraoperative complications. All three were at an advanced stage of pregnancy at the time of surgery. In two cases (C6, H8), the size of the uterus was such that the oviducts could not be adequately visualized and accessed, and the procedure was interrupted to avoid prolonged manipulations and anesthesia. The third case (S14) was a female at an advanced stage of gestation that died during the surgery for undetermined cause. In a fourth case, that was tattooed with a mark “X=,” a non-pregnant female presented abundant adhesions in the uterine zone, making it impossible to access the ovaries despite a conversion to an open laparotomy approach.

The mean surgery duration was 14 ± 3 min for non-pregnant females and 22 ± 18 min for pregnant females. The overall whole capture-release process lasted in average 3 h 22 min, and ranged from 1 h 11 min to 5 h 52 min.

#### Complications

The major complication encountered during the surgery was caused by the extent of fibrous adhesions, that significantly interfered with the exploration of the abdominal cavity. These adhesions were recorded in 24/140 (17.1%) of the treated females (Supplementary Table 1) and were mostly located in the caudal abdomen. A biopsy, obtained from a lesion appearing as a small mass located at the surface of the uterus of the patient (X=) with such adhesions and for which the laparoscopy was converted to a laparotomy, confirmed a diagnosis of endometriosis. Five cases of herniation were recorded, usually associated with dense adhesions.

### Post-operative Clinical Evaluation

The potential post-operative complications by direct observations of the released females within days after the surgery were monitored (Supplementary Figure 4). No issues apart from dehiscence of the skin suture, due to intensive scratching by the monkey, seen in 8 cases (5.7%) without major infection cues or systemic impact, no major complication and no pain-associated behavior associated with surgery in non-human primates (37, 38) have been observed after release.

To monitor the efficacy of the laparoscopic tubectomy approach, a laparoscopic post-operative assessment was made on two patients (L3, T4) randomly re-captured 1 or 2 years after their intervention. Capture, anesthesia, analgesia and laparoscopy procedures were the same as previously described. Both laparoscopic post-operative evaluations confirmed that the oviducts were definitely disrupted and no longer patent. The ovaries looked normal and were clearly no longer connected to the uterus as part of the oviduct was missing. There was no sign of local reaction or adhesions on the surgical site (Supplementary Video 3).

### Long-Term Field Monitoring

The 3-year monitoring has revealed a 100% reliability rate of the sterilization method since no new birth has been recorded so far in females which underwent surgery, and were not pregnant at that time. 96.3% of the sterilized females survived at least 6 months after the surgery and 91.9% until 3 years after surgery. This survival rate is similar to the annual survival rate of all reproductive females of this population (92.3%) calculated via a matrix population model based on 10-year life table dataset (30). Most bodies of deceased sterilized females could not be found but their behavior and condition before their disappearance suggested causes unrelated to surgery (e.g., falls from trees, human-related hazards such as electric shock in poles). Finally, the pregnancy outcome for 36 of the 40 gestating females that underwent surgery was confirmed: delivery failure (including abortion and perinatal mortality) was observed in 6 (16.7%) of these females. Relevant results are summarized in Table 1.

**Table 1:** Data summary of the treated females (*N* = 140) with frequency and proportion (%) of age class (adult, subadult), pregnancy stage at the time of the intervention, pregnancy outcome, female survival at least 6 months following surgery, and intraoperative complications.

**Parameter**	**Category**	**Occurrence**	**%**
**Age**			
	Adult	129	92.1
	Subadult	11	7.9
**Pregnancy stage**			
	Not gravid	100	71.4
	Early	11	7.9
	Mid-term	10	7.1
	Advanced	19	13.6
**Pregnancy outcome**			
	Delivery	30	83.3
	Abortion	6	16.7
	Unknown	4	10
**Survival (6 month)**			
	Yes	131	96.3
	No	5	3.7
**Intraoperative complication**			
	Tubectomy failure	4	2.9
	Adhesions	24	17.1

*For exhaustive data, see Supplementary Table 1*.

## Discussion

Contraception is increasingly used as a tool for population control of primates living in anthropogenic habitats in Asia. Despite an increasing interest in using this method, their implications and suitability remain poorly understood (39). Very few systematic studies have indeed evaluated the outputs of such measures, which constitutes a major research gap calling for robust assessments (40). This study aimed to provide an evaluation of the laparoscopic tubectomy by bipolar thermocautery as a mean of fertility control in free-ranging long-tailed macaques. It also aimed at adjusting the procedure for pregnant patients and provide a clinical evaluation of the method and its potential associated complications.

Previous studies have shown various undesirable side effects associated with castration, ovariectomy or contraceptive agents in primates (41–45). Conversely, while impeding fecundation, vasectomy and tubectomy keep intact the steroid hormonal functions underpinning normal sexual activity (20, 46). Preservation of the gonadal hormones represents the best option when sterilizing wild female macaques as it allows them to keep normal sexual and social behaviors in their group. It has also been shown to have beneficial effects on bone metabolism both in women (47) and monkeys (48). Laparoscopic tubectomy is a minimally-invasive intervention highly recommended as a substitute for conventional ovariectomy for permanent contraception of macaques as it does not induce observable hormonal alterations (49). It is associated with a lower incidence of intra- and post-operative complications (17) and significantly reduces the surgical and post-operative recovery times in primates (50). This fact is of paramount importance with social primates since prolonged separation of individuals is likely to jeopardize their reintroduction to social group. Common adverse consequences of prolonged isolation are fighting, increased aggressions toward the reintroduced animals and socio-behavioral changes including loss of the social status after reintroduction (51). The maximum holding period recommended is 24 h (52). In this study, all animals were reintroduced to their group within 6 h after capture, as reported on large cohorts (14), and the release process monitoring showed no substantial fights during release.

The technique we describe here is adapted from the endoscopic salpingectomy in wild macaques first demonstrated by P. Martelli in a DVD entitled “Endoscopic Tubectomy in Macaques” for a German company selling endoscopic equipment (Karl Storz-Endoskope) in 2009. Endoscopic salpingectomy has since widely been used for neutering campaigns in Formosan (20), rhesus (14, 17) macaques and quite recently, on a chimpanzee (*Pan troglodytes*) (53).

Here the choice of cautery, a two-port-approach, the inclusion of pregnant females and the complications as well as the modifications to the original technique are discussed.

While all previous reports combined coagulation of the oviducts followed by resection using endoscopic scissors of a section of each tube, that was removed from the abdominal cavity through the cannula (17, 20, 50, 53), this study opted for thermocautery only. The oviduct is grasped and cauterized twice but no section is resected and removed. The severed tissues are left to aseptically necrotize *in situ*, which was considered safe and sufficient to ensure sterilization of the animal as it has been shown to be as safe and effective as partial salpingectomy in women (54). Although that information is limited to two patients that underwent a second laparoscopy for control purposes, the anatomical disruption of the portion of the oviduct that had been severed by thermocautery and the consequent loss of connection between the ovary and the uterus was confirmed visually. This supports the unlikelihood of recanalization and reversal with this technique in long-tailed macaques as supported by the 100% efficacy of the method (absence of recorded pregnancy or birth) for all the patients that survived over the 3-year-period monitoring we report. These results confirm that a bipolar thermocautery alone, without further resection of the fallopian tubes is a safe and simpler approach to achieve an effective hemostasis and permanent disruption of the oviducts.

Previous reports of laparoscopic tubectomy in macaques accessed each ovary from its own side (17, 20, 50) resulting in a three-port technique. More recently, both ovaries were accessed from the same side but maintained a three-port approach (14). Although, the original plan had been to access each ovary from its own side, it became immediately obvious that the first port in the left flank could easily be used to access the right oviduct as well with the bipolar forceps, at least for non-pregnant and early to mid-term pregnant females. The two-port approach was therefore preferred as easier to perform and avoiding the opening and closing of the third access, thus reducing the duration of the procedure.

Capture is a keypoint of free-ranging animal sterilization programs. Although less stressful for the animals, and giving access to higher number of them (35), trapping in cages does not allow selection of the captured animals. Individuals that may not be contracepted have to be released with reduced chances to be trapped again in a short interval. These include females with palpable pregnancies which were not usually considered suitable candidates for surgery (14). In order to avoid having to release animals without neutering them, the technique was adjusted to allow surgery on pregnant females. The placement of the first trocar was adjusted based on gestational age and the relative position of the gravid uterus evaluated by palpation as commonly described in humans (55). While the two-port approach proved to grant easy access to both oviducts in early to mid-term pregnancies, in females nearer to their term the size of the gravid uterus precluded the adequate visualization of the contralateral oviduct. For those cases, following cautery of the left oviduct, patients were slightly tilted to the left and a third access in the right flank is performed as described above. This position is routinely recommended in human laparoscopy (55, 56) and it has been shown that it is unnecessary to place the patient in a strict lateral decubitus position (57). Not only does it allow better visualization of the right oviduct but it also reduces aortocaval compression. In humans, one of the major concerns with laparoscopy on pregnant patients was originally the effect of the CO_2_ pneumoperitoneum on the fetal physiology. However, it has been shown that no substantial adverse effects are observed when the duration of the procedure is limited (under 60 min) and the intraabdominal pressure is kept under 10–12 mmHg (58, 59). While the insufflator is set at 12 mmHg as default in this approach for non-pregnant monkeys, the intraabdominal pressure was limited as possible to 10 mmHg in advanced pregnancies as long as a sufficient visualization of the oviducts can be achieved. Accidental puncture of the gravid uterus with the Veress needle is considered the most serious complication in pregnant women (58, 60). This complication has not been observed in any of the 40 pregnant females that underwent surgery during the campaigns. It may be suspected that the size of the patients, allowing a good transabdominal palpation and localization of the uterus, permitted readjustment of the site of insertion more cranially to avoid the uterine fundus. It is worth noticing that while all the previous reports based on the initial description of the technique place the first trocar caudal to the umbilicus (14, 17, 20, 50, 53), the first access is located just cranially to the umbilicus in this study. This may contribute to an easier access to the contralateral oviduct even in pregnant patients and limit conflicts between instruments inside the abdominal cavity.

As laparoscopic oviduct cautery does not induce observable hormonal alterations (49), it is therefore not expected to interfere with pregnancy maintenance. By comparison, delivery failure was observed in 16.7% females. This proportion of abortion in gravid sterilized females was similar to or even lower than the one reported for long-tailed macaques in captivity (23.7%) (61). Out of a very large cohort, where pregnant females were normally excluded, 9 cases of tubectomy on females suspected of being pregnant based on the presence of mildly rounded congested and enlarged uteri, among which 3 completed a gestation successfully have been reported. However, as these findings were incidental and pregnancy had not been confirmed, the authors preferred not to draw conclusions (14). These results establish that the laparoscopic tubectomy did not interfere with gestation and is therefore a safe and reliable sterilization technique even for advanced pregnant female long-tailed macaques. Laparoscopic procedures for various surgical interventions during pregnancy have been considered effective and safe in women and macaques (62). Laparoscopic tubal cautery during pregnancy was also safe and feasible. However, the procedure feasibility was limited by the gestational stage: females near term (≥4 months) could not easily be treated because the size of the uterus reduced the access to oviducts. Provided that this limitation is considered, this new insight is of great interest for sterilization of animals living in free-ranging settings where captures are very challenging. That is particularly true for non-seasonal breeding species, where no optimal timing of capture can be identified because of year-round distribution of births. Captures can therefore be optimized if most of the gestating females can be included and undergo tubectomy.

Complications of endoscopic salpingectomy, including difficult entry into the abdominal cavity using the Veress needle, intraoperative hemorrhage and inadvertent cauterization of the uterus have been reported in Formosan macaques (20). As in other reports (14), very few intraoperative complications were observed during this study's campaigns. The main intraoperative limitation was due to the presence of adhesions, observed in 24/140 (17%) of the operated females out of which 16 were not pregnant. In one case, these adhesions were such that access to the ovaries could not be achieved even after conversion to a standard laparotomy. A biopsy obtained from this patient confirmed the presumptive diagnosis of endometriosis associated with these cases of adhesions mainly located in the caudal abdomen. The incidence of spontaneous endometriosis in a breeding colony of *Macaca fascicularis* has been reported to be 28.7% (63) in aging females. Lesions of endometriosis have recently been characterized in long-tailed macaques and involved fibrous adhesions, especially between the omentum and the uterus, liver, spleen, and urinary bladder (64). The same paper also describes cases where one or both ovaries could not be identified and were suspected to be incorporated or effaced by the fibrous adhesions. Although, it would be hazardous to conclude that all cases with intra-abdominal adhesions would necessarily be due to endometriosis, this is exactly what was observed in the patient [“(X=)” where endometriosis was confirmed. In non-human primates, decidualization of endometriotic tissue most often occurs under the influence of exogenous progestins or during pregnancy (65). In this study, none of the 24 animals with such lesions received exogenous progestins and 16 were not pregnant, supporting the hypothesis of that endometriotic tissue may decidualize under the influence of elevated endogenous progesterone (64).

Post-operatively, 8 cases of partial wound dehiscence were observed which did not impact the general condition of the animals and was self-limited. These delayed wound healing came unsurprisingly as grooming behavior, wound, or stitching material picking are common in macaques (66). However, this complication has not been mentioned in other studies (17) and more particularly in a large scale study (14) where the skin was closed using surgical glue rather than absorbable suturing material, which should be considered to further improve our technique. No other complications were recorded. Systematic post-operative observations are not feasible with animals living in a semi-wild habitat, and although no pain scoring observations were performed, females that were seen in the days following surgery showed no apparent signs of pain and displayed normal behavior of feeding and grooming. These observations support the fact that, laparoscopic tubectomy being a minor surgical procedure, one single administration of NSAIDs for analgesia was sufficient to allow swift release of the animals back to their social groups.

Although this duration may need to be reduced for smaller animals (<2 kg) because of their propensity to hypoglycemia, it is usually considered advisable to withhold food for 12–16 h and water for 2 h before induction of anesthesia of non-human primates (67). This reduces the risk of gastrointestinal bloat and aspiration pneumonia as macaques may vomit on induction of anesthesia. Fasting the animals—or even administration of metoclopramide 30–60 min before induction which is also sometimes recommended (67)—was unfortunately not possible under the conditions of this study. However, like others (14), no such complications were observed during our sterilization campaigns and thorough emptying of the cheek pouches was considered sufficient to prevent aspiration pneumonia.

In this study, the mean surgery duration of the laparoscopic tubectomy was 14 ± 3 min for non-pregnant females and 22 ± 18 min for pregnant females. This is longer than the 3–10 min reported in other papers (14, 17) that included non-pregnant females only. Possible explanations for this difference may be found in the degree of experience of the operator but also in at least two technical aspects. First, females with pre-existing severe adhesions that sometimes seriously interfered with the access to the oviducts were included and thus prolonged the duration of the procedure. Second, 5 mm trocars instead of 3 mm ones were used, which led to suture the abdominal wall rather than leaving it unsutured. Finally, to limit wound picking, the skin was apposed with an intradermal suture instead of surgical glue which is obviously faster. Laparoscopic vessel sealant tools, not yet available in 3 mm, were shown not necessary and that standard bipolar forceps, that are available both in 3- and 5-millimeter size, were sufficient to efficiently sterilize the patients. Smaller size instruments should therefore probably be preferred for surgeries on animals the size of a macaque.

As a conclusion, laparoscopic oviduct cautery with standard bipolar forceps can be adjusted to safely sterilize barren and pregnant long-tailed macaques even at late stages of pregnancy. Use of a single lateral port is feasible in non-gestating and early to mid-term pregnant females, while females nearer to their term are best operated using a three-port entry. The idea that including pregnant females and patients with some degree of adhesions will optimize the trapping efforts and the overall success of population control programs. The absence of significant complications with the technique, which allows release of free-ranging animals very shortly after procedure and avoids complications related to prolonged isolation from the group. The technique could be further optimized by combining a 3 mm bipolar forceps via a two-port approach and the use of surgical glue for the closure of the skin, as minuscule accesses allow suture of the linea alba only thus making the entire procedure shorter again.

Our follow-up over a 3-year period on patients' clinical evolution, pregnancy outcome, and later infertility demonstrates the safety and efficiency of the modifications brought to the procedure. Moreover, as controlling effects of fertility control on wild animal life is of paramount importance, a long-term monitoring of this population is still ongoing and further focuses on the potential impact of reproduction suppression on behavior and social dynamics (68). Effects of reproduction suppression on the health, welfare, social behavior, and ranging patterns of primates are often only superficially considered. This influence needs to be examined to further complete the data on the technique and its impacts beyond the population control aspects.

## Data Availability Statement

The original contributions presented in the study are included in the article/Supplementary Material, further inquiries can be directed to the corresponding author/s.

## Ethics Statement

The whole protocol of this study followed the ethical recommendations for animal welfare (36). All procedures have been chosen in order to cause the least stress, pain and discomfort to the monkeys (e.g., collective trapping allowing to achieve a maximal number of captures while causing the least stress and discomfort to the monkeys, limiting handling when animals are not sedated, minimally invasive endoscopic tubectomy procedure, administration of NSAID for analgesia and a holding period as short as possible). This research has been approved by the Animal Ethics Commission of Udayana University (No. 282/KE-PH/I/2017) and has been granted with permission of the Provincial Office of Conservation in Bali (BKSDA, No. KT49/BKSDA BL-1/KK/2017; Capture license: #29/PPSP/XII/2017). Moreover, this research was conducted under research permission from the Indonesian Ministry of Research and Technology (No. 186/SIP/FRP/E5/Dit.KI/VII/2017, No. 46/SIP/FRP/E5/Dit.KI/II/ 2018, No. 10/E5/E5.4/ SIP.EXT/2019, No. 83/E5/E5.4/SIP.EXT/2019). Universal precautions (gloves, masks, and overshoes/caps specifically for surgery) against hazards and zoonotic pathogen transmission were carefully taken during the whole process from capture, clinical exam, surgery and postoperative procedures.

## Author Contributions

FB, SD, and M-CH: conceived and designed the study. SD, RP, and GS: undertook the laparoscopic tubectomy and collected clinical data. SD, GR, M-CH, FB, NW, GS, GG, and FC: veterinary care and logistics during the sterilization campaigns. GG, FB, and FC: data collection for field monitoring. FB: data analyses. NW and PP: facilitation to fieldwork. SD and FB: wrote the manuscript. M-CH: provided in-depth editorial advice. All authors reviewed and edited the manuscript and approved the submitted version.

## Funding

This study was carried out with the financial support of the Padangtegal village leadership committee, the Belgian National Fund for Scientific Research, the Erasmus + International Credit Mobility Programme, the University of Liège, and Wallonie-Bruxelles International.

## Conflict of Interest

The authors declare that the research was conducted in the absence of any commercial or financial relationships that could be construed as a potential conflict of interest.

## Publisher's Note

All claims expressed in this article are solely those of the authors and do not necessarily represent those of their affiliated organizations, or those of the publisher, the editors and the reviewers. Any product that may be evaluated in this article, or claim that may be made by its manufacturer, is not guaranteed or endorsed by the publisher.
