# Supplementary material for: Tubectomy of Pregnant and Non-pregnant Female Balinese Macaques (Macaca Fascicularis) With Post-operative Monitoring
Source: Front Vet Sci. 2021 Sep 9;8:688656. doi: 10.3389/fvets.2021.688656 (PMC8458650; doi:10.3389/fvets.2021.688656)
Supplement: Supplementary file 4 [file Data_Sheet_1.docx]

**Supplementary Material**

**Supplementary Table S1.** List of the treated females (*N* = 140) with for each of them: identity (tattoo ID), group, age class (A: adult, S: subadult), body weight (kg), date of intervention, pregnancy stage at the time of the intervention, pregnancy outcome, female survival at least 6 months following surgery, clinical remarks related to surgery and follow-up notes.

NA = data not available

| **N°** | **Tattoo ID** | **Group** | **Age class** | **Weight** | **Date of surgery** | **Pregnancy stage** | **Pregnancy outcome** | **Female survival 6 mo** | **Surgery clinical remark** | **Follow-up notes** |
| --- | --- | --- | --- | --- | --- | --- | --- | --- | --- | --- |
| 1 | H1 | H | A | 5.2 | 2017-07-30 |  |  | Yes |  |  |
| 2 | H2 | H | A | 4.2 | 2017-07-30 |  |  | Yes |  | Found dead (June 2018, fight) |
| 3 | C1 | C | A | 5.0 | 2017-07-30 | Mid-term | Delivery | Yes |  |  |
| 4 | C2 | C | A | 6.4 | 2017-07-30 |  |  | Yes |  |  |
| 5 | C3 | C | A | 6.1 | 2017-07-30 | Early | Abortion | Yes |  |  |
| 6 | C4 | L | A | 4.5 | 2017-07-30 |  |  | Yes |  |  |
| 7 | T1 | T | A | 5.0 | 2017-07-30 |  |  | Yes |  |  |
| 8 | T4 | T | A | 6.4 | 2017-08-01 | Early | Delivery | Yes |  | Laparoscopic follow-up case #1 (recaptured in July 2018) |
| 9 | T5 | T | A | 5.1 | 2017-08-01 |  |  | Yes | Adhesions (abdomen) |  |
| 10 | T6 | T | A | 5.4 | 2017-08-01 |  |  | Yes | Adhesions (abdomen) |  |
| 11 | N2 | N | A | 5.2 | 2017-08-01 |  |  | Yes |  |  |
| 12 | N3 | N | A | 5.4 | 2017-08-01 |  |  | Yes | Adhesions (abdomen), hernia |  |
| 13 | N4 | N | A | 4.5 | 2017-08-01 |  |  | Yes |  |  |
| 14 | C5 | C | A | 6.5 | 2017-08-01 |  |  | Yes |  |  |
| 15 | X1 | X | A | 4.8 | 2017-08-01 |  |  | Yes |  |  |
| 16 | T7 | T | A | 5.8 | 2017-08-03 |  |  | Yes |  |  |
| 17 | T8 | T | A | 5.7 | 2017-08-03 |  |  | Yes | Adhesions (omentum, abdomen) |  |
| 18 | T9 | T | A | 4.2 | 2017-08-03 |  |  | Yes |  |  |
| 19 | T10 | T | A | 4.2 | 2017-08-03 |  |  | Yes |  |  |
| 20 | L3 | L | A | 4.4 | 2017-08-03 |  |  | Yes |  | Laparoscopic follow-up case #2 (recaptured in Feb 2019) |
| 21 | L4 | L | A | 6.2 | 2017-08-03 |  |  | Yes |  |  |
| 22 | L6 | L | A | 5.5 | 2017-08-03 |  |  | Yes |  |  |
| 23 | T11 | X | A | 5.2 | 2018-07-22 | Early | NA | No |  | Disappeared (Nov 2018) |
| 24 | T12 | X | A | 5.0 | 2018-07-22 |  |  | Yes | Adhesions (fallopian tubes) |  |
| 25 | X4 | X | A | 4.6 | 2018-07-22 | Early | Delivery | Yes |  |  |
| 26 | X5 | X | A | 6.1 | 2018-07-22 |  |  | Yes |  |  |
| 277 | X6 | X | A | 5.4 | 2018-07-22 |  |  | Yes |  |  |
| 28 | X7 | X | A | 4.5 | 2018-07-25 |  |  | Yes |  |  |
| 29 | L8 | L | A | 6.7 | 2018-07-25 |  |  | Yes |  |  |
| 30 | L9 | L | A | 5.8 | 2018-07-25 |  |  | Yes |  |  |
| 31 | L10 | L | A | 6.2 | 2018-07-25 |  |  | Yes | Adhesions (abdomen) and abdominal hernia |  |
| 32 | L12 | L | A | 4.6 | 2018-07-25 |  |  | Yes |  |  |
| 33 | S2 | S | A | 4.8 | 2018-07-25 |  |  | Yes |  |  |
| 34 | S3 | S | A | 5.2 | 2018-07-25 | Early | Delivery | Yes |  |  |
| 35 | X= | X | A | 7.0 | 2018-07-28 |  |  | Yes | Laparotomy. Tubectomy failure due to massive adhesions and umbilical hernia | Not sterilized, probably naturally sterile due to endometriosis |
| 36 | X8 | X | A | 5.3 | 2018-07-28 |  |  | Yes |  |  |
| 37 | X10 | X | A | 3.9 | 2018-07-28 | Early | NA | Yes |  |  |
| 38 | X11 | X | A | 5.5 | 2018-07-28 |  |  | Yes |  |  |
| 39 | X13 | X | A | 4.4 | 2018-07-28 |  |  | Yes |  |  |
| 40 | X14 | X | A | 4.1 | 2018-07-28 |  |  | Yes |  |  |
| 41 | H5 | H | A | 4.3 | 2018-07-28 |  |  | Yes |  |  |
| 42 | L13 | L | A | 4.5 | 2018-07-28 |  |  | Yes |  |  |
| 43 | L14 | L | A | 4.3 | 2019-02-02 |  |  | No | Cataract | Disappeared (Apr 2019) |
| 44 | L15 | L | A | 7.1 | 2019-02-02 |  |  | Yes | Adhesions (umbilicus) |  |
| 45 | L16 | L | A | 3.4 | 2019-02-02 |  |  | Yes |  |  |
| 46 | L17 | L | S | 5.1 | 2019-02-02 |  |  | Yes |  |  |
| 47 | L19 | L | A | 7.1 | 2019-02-02 |  |  | Yes |  |  |
| 48 | L20 | L | A | 4.8 | 2019-02-02 |  |  | Yes |  |  |
| 49 | L21 | L | A | 3.4 | 2019-02-02 |  |  | Yes |  |  |
| 50 | T13 | T | A | 4.9 | 2019-02-04 |  |  | Yes |  |  |
| 51 | T14 | T | A | 5.6 | 2019-02-04 | Advanced | Delivery | Yes |  |  |
| 52 | T15 | T | A | 5.2 | 2019-02-04 |  |  | Yes |  |  |
| 53 | T16 | T | A | 7.5 | 2019-02-04 | Mid-term | Delivery | Yes |  |  |
| 54 | T17 | T | A | 5.5 | 2019-02-04 | Advanced | Delivery | Yes |  |  |
| 55 | T18 | T | A | 3.8 | 2019-02-04 |  |  | Yes |  |  |
| 56 | T19 | T | A | 5.8 | 2019-02-04 | Advanced | Delivery | Yes |  |  |
| 57 | T20 | T | S | 3.0 | 2019-02-04 |  |  | Yes |  |  |
| 58 | T21 | T | A | 5.3 | 2019-02-04 | Advanced | Delivery | Yes |  |  |
| 59 | T22 | T | A | 4.5 | 2019-02-04 |  |  | Yes |  |  |
| 60 | T23 | T | A | 4.7 | 2019-02-04 |  |  | Yes |  |  |
| 61 | T24 | T | A | 4.5 | 2019-02-04 |  |  | Yes |  |  |
| 62 | S5 | S | A | 5.2 | 2019-02-07 |  |  | Yes |  |  |
| 63 | S6 | S | A | 6.5 | 2019-02-07 |  |  | Yes |  |  |
| 64 | S7 | S | A | 6.1 | 2019-02-07 |  |  | Yes |  |  |
| 65 | S8 | S | A | 5.7 | 2019-02-07 |  |  | Yes | Adhesions (abdomen wall) |  |
| 66 | S9 | S | A | 6.2 | 2019-02-07 |  |  | Yes |  |  |
| 67 | S10 | S | A | 5.5 | 2019-02-07 | Advanced | Abortion | Yes |  |  |
| 68 | S11 | S | A | 6.0 | 2019-02-07 |  |  | Yes |  |  |
| 69 | S12 | S | A | 4.1 | 2019-02-07 | Mid-term | Delivery | Yes | Adhesions (bladder, ovaries) |  |
| 70 | H6 | H | A | 6.0 | 2019-02-07 | Mid-term | Abortion | Yes |  |  |
| 71 | H7 | H | A | 7.5 | 2019-02-07 |  |  | Yes |  |  |
| 72 | H8 | H | A | 5.8 | 2019-02-10 | Mid-term | Delivery | Yes | Tubectomy failure due to massive adhesions (bladder, ovary) and big uterus size: right ovary not visualized | Not sterilized |
| 73 | X17 | X | A | 4.5 | 2019-02-10 |  |  | Yes |  |  |
| 74 | X18 | X | A | 4.7 | 2019-02-10 |  |  | Yes |  |  |
| 75 | X19 | X | A | 6.0 | 2019-02-10 |  |  | Yes |  |  |
| 76 | X20 | X | A | 4.3 | 2019-02-10 |  |  | Yes |  |  |
| 77 | X21 | X | S | 3.5 | 2019-02-10 |  |  | Yes |  |  |
| 78 | X22 | X | A | 8.3 | 2019-02-10 | Advanced | Delivery | Yes |  |  |
| 79 | X23 | X | S | 3.8 | 2019-02-10 |  |  | Yes |  |  |
| 80 | H9 | H | A | 4.6 | 2019-02-10 | Early | Abortion | Yes | Adhesions (intestine) and inguinal hernia |  |
| 81 | H10 | H | A | 4.2 | 2019-02-10 | Advanced | Delivery | Yes |  |  |
| 82 | N5 | N | A | 6.2 | 2019-02-14 |  |  | Yes |  |  |
| 83 | N6 | N | A | 4.7 | 2019-02-14 | Early | Delivery | Yes |  |  |
| 84 | N7 | N | A | 5.0 | 2019-02-14 | Advanced | Abortion | Yes |  |  |
| 85 | N8 | N | A | 6.1 | 2019-02-14 | Advanced | Delivery | Yes | Adhesions (uterus, right ovary, abdomen wall) |  |
| 86 | N9 | N | A | 3.8 | 2019-02-14 |  |  | Yes |  |  |
| 87 | N10 | N | A | 4.9 | 2019-02-14 |  |  | Yes |  |  |
| 88 | N11 | N | A | 4.7 | 2019-02-14 | Advanced | Delivery | Yes |  |  |
| 89 | H3 | H | A | 7.4 | 2019-02-14 |  |  | Yes |  |  |
| 90 | H4 | H | A | 4.6 | 2019-02-14 |  |  | Yes |  |  |
| 91 | H11 | H | A | 7.3 | 2019-02-14 |  |  | Yes | Adhesions (ovary) |  |
| 92 | C6 | C | A | 5.9 | 2019-02-14 | Advanced | Delivery | Yes | Tubectomy failure due to advanced pregnancy: uterus size impeded the visualization of the right oviduct | Not sterilized |
| 93 | S13 | S | A | 6.4 | 2019-08-01 |  |  | Yes |  |  |
| 94 | S14 | S | A | 6.9 | 2019-08-01 | Advanced | NA | No | Dead during surgery (undetermined cause), adhesions (right flank) | Dead during surgery |
| 95 | S15 | S | A | 5.9 | 2019-08-01 |  |  | Yes | Adhesions (median septum) |  |
| 96 | S16 | S | A | 5.0 | 2019-08-01 |  |  | Yes |  |  |
| 97 | S17 | S | S | 4.3 | 2019-08-01 |  |  | Yes | Adhesions (bladder) |  |
| 98 | S18 | S | A | 3.6 | 2019-08-01 |  |  | Yes |  |  |
| 99 | H12 | H | A | 3.5 | 2019-08-01 |  |  | Yes | Adhesions (ovaries) |  |
| 100 | N12 | N | S | 3.4 | 2019-08-01 |  |  | Yes |  |  |
| 101 | L22 | L | A | 4.6 | 2019-08-04 |  |  | Yes |  |  |
| 102 | L23 | L | A | 6.4 | 2019-08-04 | Mid-term | Delivery | Yes |  |  |
| 103 | L24 | L | A | 5.4 | 2019-08-04 | Early | Delivery | Yes |  |  |
| 104 | L25 | L | A | 4.5 | 2019-08-04 |  |  | Yes |  |  |
| 105 | L26 | L | A | 4.8 | 2019-08-04 | NA | NA | Yes |  |  |
| 106 | H13 | H | A | 5.0 | 2019-08-04 |  |  | Yes | Adhesions (uterus, right flank) |  |
| 107 | H14 | H | A | 5.0 | 2019-08-04 |  |  | Yes |  |  |
| 108 | H15 | H | S | 3.5 | 2019-08-04 |  |  | Yes | Adhesions (abdomen) |  |
| 109 | H16 | H | A | 5.3 | 2019-08-04 |  |  | Yes |  |  |
| 110 | C7 | C | A | 6.5 | 2019-08-04 | Advanced | Delivery | Yes |  |  |
| 111 | T25 | T | A | 5.5 | 2019-08-07 |  |  | Yes |  |  |
| 112 | T26 | T | A | 6.6 | 2019-08-07 | Advanced | Delivery | Yes |  |  |
| 113 | T27 | T | A | 4.7 | 2019-08-07 |  |  | Yes |  |  |
| 114 | T28 | T | A | 6.4 | 2019-08-07 | Advanced | Delivery | Yes | Adhesions (abdomen) |  |
| 115 | T29 | T | S | 3.7 | 2019-08-07 |  |  | Yes |  |  |
| 116 | C8 | C | A | 4.5 | 2019-08-07 |  |  | Yes |  |  |
| 117 | C9 | C | A | 5.9 | 2019-08-07 | Early | Delivery | Yes | Adhesions, umbilical hernia |  |
| 118 | S19 | S | A | 4.1 | 2019-08-07 |  |  | Yes |  |  |
| 119 | S20 | S | A | 4.1 | 2019-08-07 | Mid-term | Delivery | Yes | Adhesions (abdomen) |  |
| 120 | S21 | S | A | 4.5 | 2019-08-07 |  |  | Yes |  |  |
| 121 | S22 | S | A | 4.0 | 2019-08-07 |  |  | Yes |  |  |
| 122 | T30 | T | A | 4.9 | 2019-08-10 |  |  | Yes |  |  |
| 123 | T31 | T | A | 5.0 | 2019-08-10 |  |  | Yes |  |  |
| 124 | T32 | T | A | 6.1 | 2019-08-10 | Advanced | Delivery | Yes |  |  |
| 125 | T33 | T | S | 3.8 | 2019-08-10 |  |  | Yes |  |  |
| 126 | T34 | T | A | 5.2 | 2019-08-10 | Mid-term | Delivery | Yes |  |  |
| 127 | T35 | T | A | 5.0 | 2019-08-10 |  |  | Yes |  |  |
| 128 | H17 | H | S | 4.5 | 2019-08-10 |  |  | Yes | Adhesions (abdomen) |  |
| 129 | L27 | L | A | 6.1 | 2019-08-10 | Advanced | Delivery | Yes |  |  |
| 130 | L28 | L | A | 5.0 | 2019-08-10 | Mid-term | Abortion | Yes |  |  |
| 131 | N13 | N | A | 4.5 | 2019-08-10 | Mid-term | Delivery | Yes |  |  |
| 132 | X24 | X | A | 5.1 | 2019-08-10 | Advanced | Delivery | Yes |  |  |
| 133 | X25 | X | A | 7.1 | 2019-08-12 |  |  | Yes |  |  |
| 134 | X26 | X | A | 7.0 | 2019-08-12 | Advanced | Delivery | Yes |  |  |
| 135 | L30 | L | A | 8.5 | 2019-08-12 |  |  | No |  | Disappeared (Aug 2019) |
| 136 | T36 | T | A | 4.5 | 2019-08-12 |  |  | Yes |  |  |
| 137 | T37 | T | A | 6.1 | 2019-08-12 |  |  | Yes |  |  |
| 138 | T38 | T | A | 5.5 | 2019-08-12 |  |  | No |  | Recaptured (Aug 2019) following major arm injuries (fight). Dead (2019-08-22) |
| 139 | C10 | C | A | 5.8 | 2019-08-12 |  |  | No |  | Disappeared (Sept 2019) |
| 140 | H18 | H | S | 5.9 | 2019-08-12 |  |  | Yes |  |  |

**
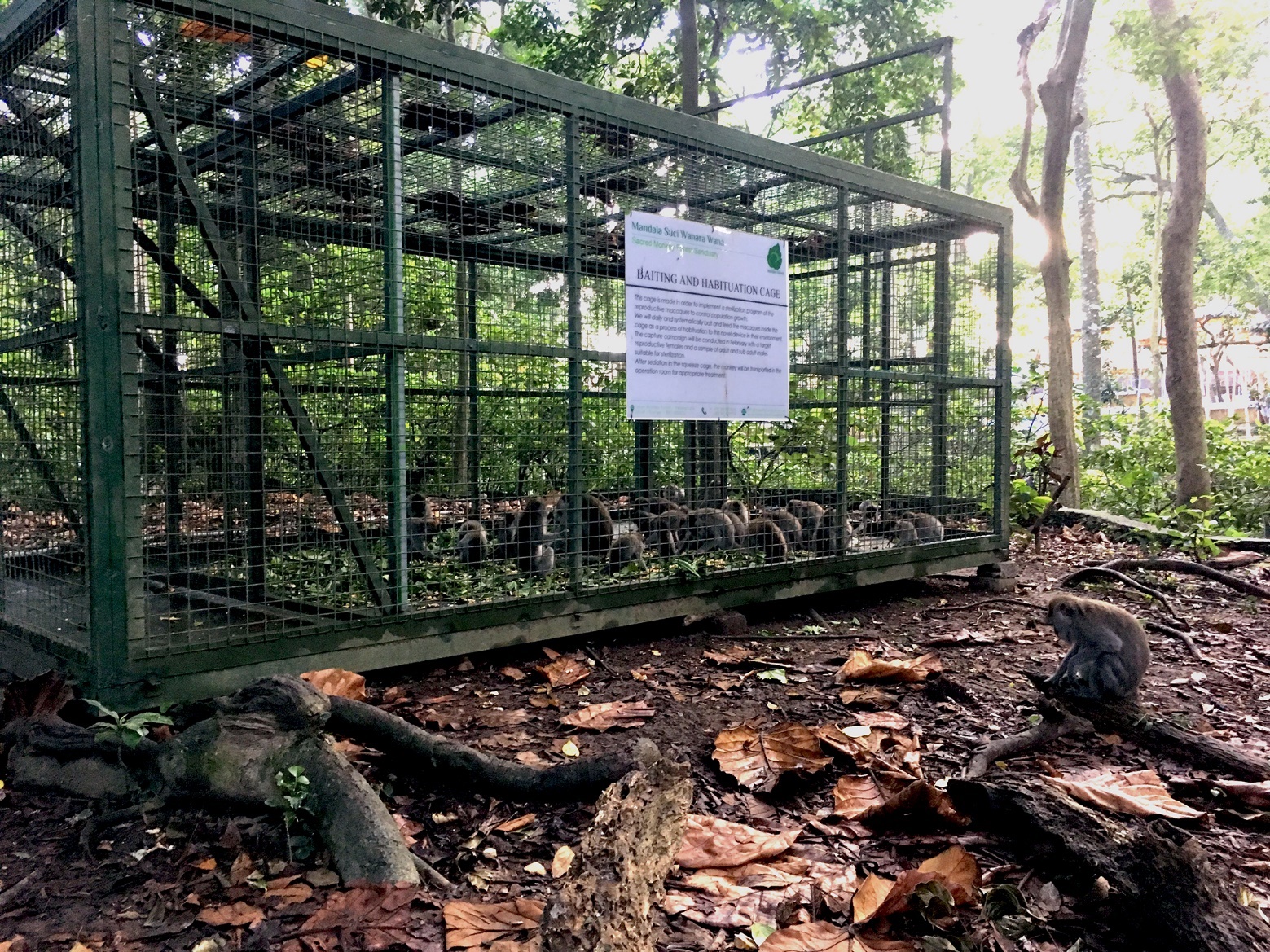
Supplementary Figure S1.** Habituation process by food-baiting at one of the trapping cages in the Ubud Monkey Forest

**Supplementary Figure S2.**
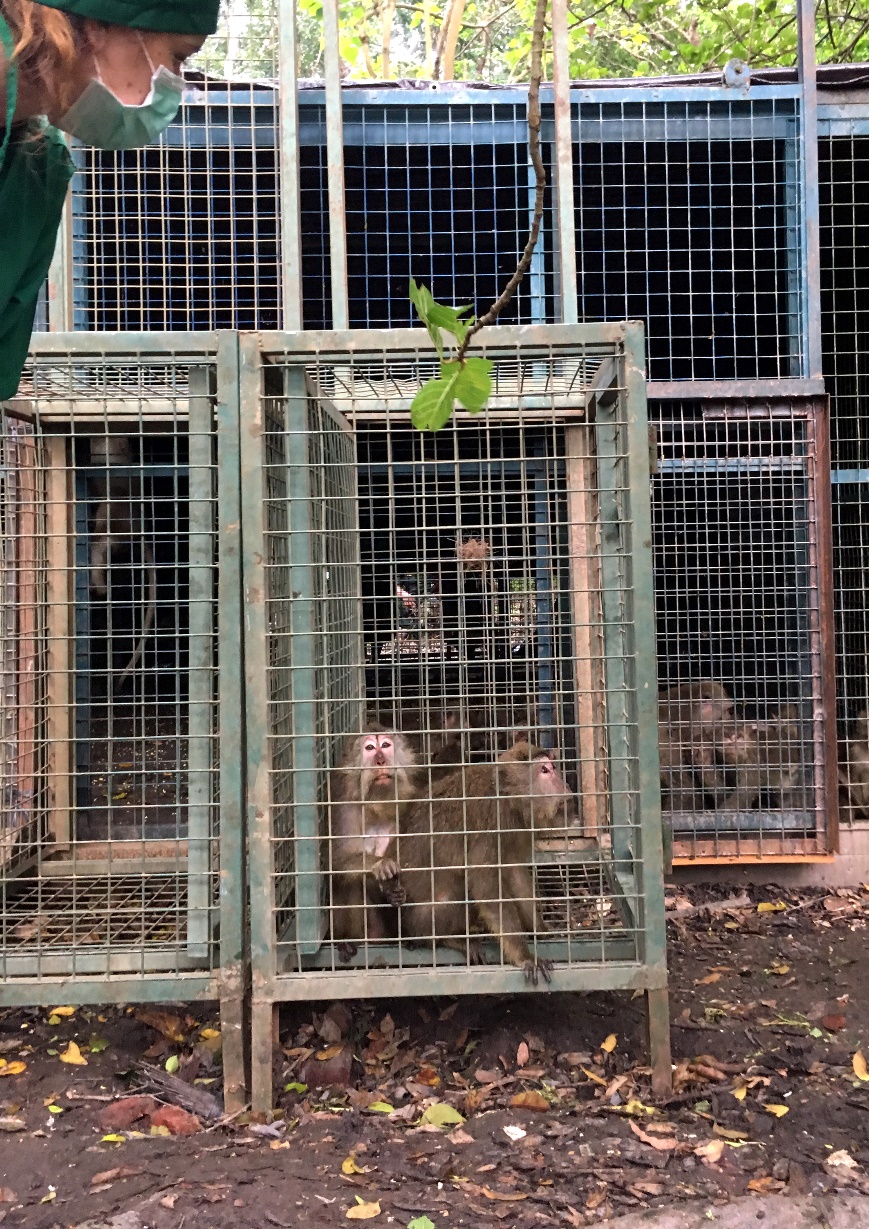
Macaques before anesthesia in a squeeze cage connected to a trapping cage


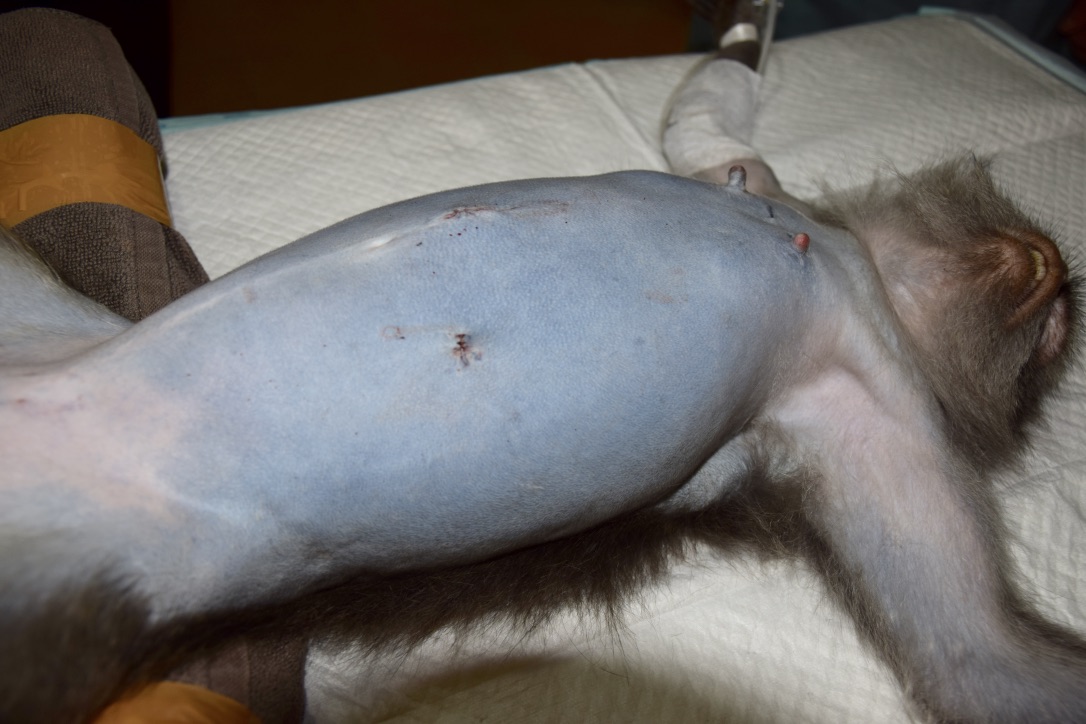


**Supplementary Figure S3**. Position of the central (cranially to the umbilicus) and lateral (left lower quadrant, cranially to the oviduct) sutured incisions following endoscopic tubectomy


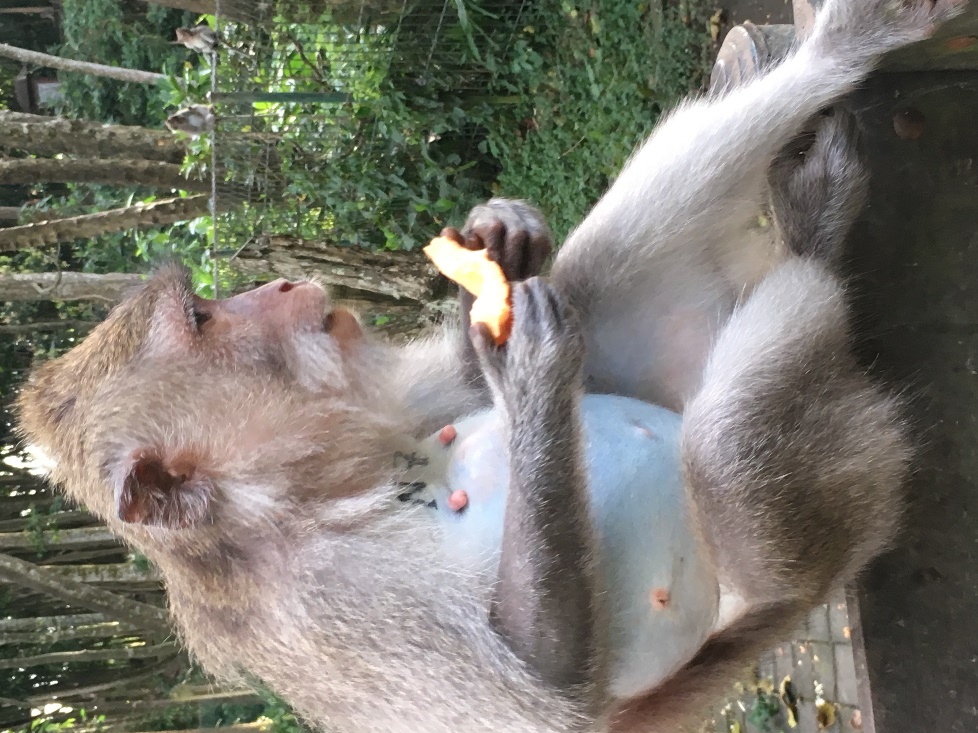


**Supplementary Figure S4.** Marking (tattoo on the chest) and healing process of the sutures in a pregnant female sterilized at the Ubud Monkey Forest


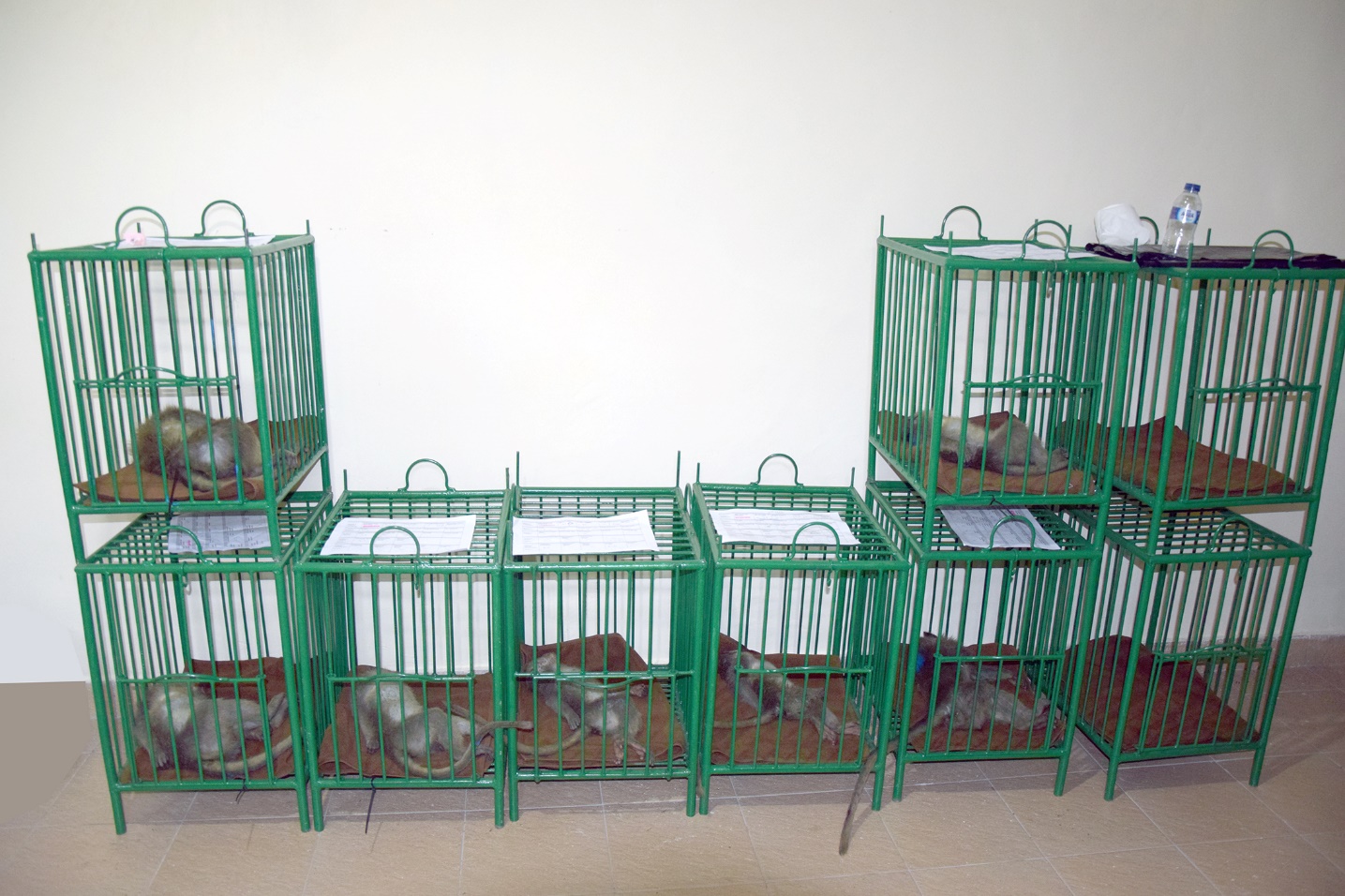


**Supplementary Figure S5.** Macaques were positioned in lateral recumbency in individual holding cages for recovery before release
